# Vitamin B12 Attenuates Changes in Phospholipid Levels Related to Oxidative Stress in SH-SY5Y Cells

**DOI:** 10.3390/cells11162574

**Published:** 2022-08-18

**Authors:** Elena Leoni Theiss, Lea Victoria Griebsch, Anna Andrea Lauer, Daniel Janitschke, Vincent Konrad Johannes Erhardt, Elodie Christiane Haas, Konstantin Nicolas Kuppler, Juliane Radermacher, Oliver Walzer, Dorothea Portius, Heike Sabine Grimm, Tobias Hartmann, Marcus Otto Walter Grimm

**Affiliations:** 1 Experimental Neurology, Saarland University, 66424 Homburg, Germany; 2 Nutrition Therapy and Counseling, Campus Gera, SRH University of Applied Health Science, 07548 Gera, Germany; 3 Deutsches Institut für DemenzPrävention, Saarland University, 66424 Homburg, Germany; 4 Nutrition Therapy and Counseling, Campus Rheinland, SRH University of Applied Health Sciences, 51377 Leverkusen, Germany

**Keywords:** oxidative stress, vitamin B12 deficiency, lipidomics, Alzheimer´s disease, anti-oxidants, phospholipids, plasmalogens

## Abstract

Oxidative stress is closely linked to Alzheimer’s disease (AD), and is detected peripherally as well as in AD-vulnerable brain regions. Oxidative stress results from an imbalance between the generation and degradation of reactive oxidative species (ROS), leading to the oxidation of proteins, nucleic acids, and lipids. Extensive lipid changes have been found in post mortem AD brain tissue; these changes include the levels of total phospholipids, sphingomyelin, and ceramide, as well as plasmalogens, which are highly susceptible to oxidation because of their vinyl ether bond at the sn-1 position of the glycerol-backbone. Several lines of evidence indicate that a deficiency in the neurotropic vitamin B12 is linked with AD. In the present study, treatment of the neuroblastoma cell line SH-SY5Y with vitamin B12 resulted in elevated levels of phosphatidylcholine, phosphatidylethanolamine, sphingomyelin, and plasmalogens. Vitamin B12 also protected plasmalogens from hydrogen peroxide (H_2_O_2_)-induced oxidative stress due to an elevated expression of the ROS-degrading enzymes superoxide-dismutase (SOD) and catalase (CAT). Furthermore, vitamin B12 elevates plasmalogen synthesis by increasing the expression of alkylglycerone phosphate synthase (AGPS) and choline phosphotransferase 1 (CHPT1) in SH-SY5Y cells exposed to H_2_O_2_-induced oxidative stress.

## 1. Introduction

Alzheimer´s disease (AD) is a neurodegenerative disease and the most common cause of dementia in individuals aged 65 years or older. Dementia, also called major neurocognitive disorder, currently affects about 55 million people worldwide, a large percentage of whom are undiagnosed, especially in lower-income countries [1]. Typical histopathological hallmarks of AD have been described in brain areas such as the hippocampus or cortex, and include intracellular neurofibrillary tangles caused by hyperphosphorylated tau proteins, or extracellular plaques caused by an oligomerization of the neurotoxic peptide amyloid-β (Aβ). AD is a multifactorial disorder and, in line with this, multiple risk factors have already been identified. For the familial form of AD, which accounts for only 5% of all cases, mutations of genes involved in the generation of Aβ are known to be genetic risk factors. In the case of the common sporadic form of AD, several factors besides age, such as obesity, hypercholesterolemia, hypertension, or hyperhomocysteinemia, have been shown to increase the risk for the development of AD [2].

On the molecular level, the occurrence of oxidative stress is another risk factor for AD as it is involved in the pathogenesis of this disorder. Hippocampal neurons are, in general, highly vulnerable to oxidative stress and require a large pool of the free-radical-eliminating antioxidant glutathione, due to their persistent ROS production [3]. Moreover, lipid peroxidation, a non-enzymatic process which is caused by reactive oxygen species (ROS), mainly hydroxyl radical (HO) and hydroperoxyl (HO_2_) [4], makes a significant contribution to the pathogenesis of AD, since processes leading to the generation of Aβ take place on and in biological membranes. The incorporation of high amounts of polyunsaturated fatty acids (PUFAs) in the phospholipids of neuronal membranes may be a risk factor for the presence of oxidative stress, since ω-3 PUFAs are especially susceptible to lipid peroxidation due to their high number of double bonds [5]. In this context, it has been demonstrated that just 1% of oxidized docosahexaenoic acid (DHA), a PUFA beneficially associated with Aβ production, and thereby with the risk of AD, diminishes its protective effect [6]. Additional evidence for a link between oxidative stress and AD comes from a study that analyzed human post mortem brain samples, showing significantly elevated levels of oxidized lipids and 4-hydroxy-nonenal (HNE) in AD brain samples, compared to samples from non-demented control individuals. Moreover, the investigated oxidized lipid species and lipid peroxidation products (HNE and 4-hydroxy-hexanal, HHE) increased the levels of Aβ in the included cell culture analysis, thereby accelerating the onset of AD [6]. Plasmalogens represent a further AD-related lipid species that is highly sensitive to ROS. Due to their vinyl ether bond, plasmalogens are susceptible to singlet oxygen and hydroxyl radicals, and are considered to be antioxidant molecules [7]. Plasmalogens are essential for brain function as they are structural components of, among others, myelin or synaptic membranes; moreover, deficits in brain plasmalogens are closely associated with AD [8].

Based on these findings, the potential of antioxidants to be used in the prevention or the treatment of AD is the subject of the current research. In this context, several lines of evidence indicate the anti-oxidative properties of vitamin B12 (cobalamin) and associate hypovitaminosis with oxidative stress. These anti-oxidative properties can either be direct, by ROS scavenging in cytosol and mitochondria, or indirect, by preserving glutathione [9,10]. Moreover, vitamin B12 has been reported to modulate the production of cytokines (interleukin-6 or tumor necrosis factor alpha) and growth factors (epidermal growth factor) and thereby to protect against inflammation-induced oxidative stress [11,12]. Additionally, the essential and water-soluble vitamin B12 beneficially influences homocysteine-induced oxidative stress, as it is an important co-factor of methionine synthase, which converts homocysteine to methionine. Accordingly, deficits in vitamin B12 result in increased intracellular homocysteine levels mediating an accumulation of ROS. This is caused either by the generation of H_2_O_2_ due to the auto-oxidation of homocysteine, or by the inhibition of cellular antioxidant enzymes, such as glutathione peroxidase and superoxide dismutase [13]. In addition to this association with oxidative stress, vitamin B12 is described as being involved in many metabolic pathways: in particular, amino acid metabolism, DNA synthesis, and fatty acid metabolism [14].

As previously discussed, one of the most important risk factors for the development and progression of AD is aging; moreover, most very elderly individuals are affected by a vitamin B12 deficiency [15]. This hypovitaminosis in the elderly could be due to an age-related decline in stomach acidity and/or an insufficient dietary uptake of vitamin B12 [16]. In line with this, a causal link between vitamin B12 deficiency and an increased risk of neurodegenerative disorders, such as AD, is homogenously described in the literature [17,18,19,20,21].

The aim of this study was to analyze whether vitamin B12 is able to protect AD-relevant lipids by decreasing oxidative-stress-mediated changes, such as lipid peroxidation. Therefore, a shotgun lipidomics approach of SH-SY5Y neuroblastoma cells treated with vitamin B12 was used to analyze changes in the lipid homeostasis.

## 2. Materials and Methods

### 2.1. Chemicals and Standards

All chemicals used in this study were purchased from Fisher Scientific (Schwerte, Germany), unless stated otherwise. High performance liquid chromatography (HPLC)-grade cobalamin and pyridine, and ammonium acetate and phenyl isothiocyanate were acquired from Merck (Darmstadt, Germany). The standards used for normalization in the lipid analysis were purchased from Supelco Analytical (Darmstadt, Germany) (octanoyl-L-carnitine d_3_, palmitoyl-L-carnitine d_3_) or from Avanti Polar Lipids (Alabaster, AL, USA) (06:0 PC (DHPC), 19:0 Lyso-PC, C18(Plasm)-18:1(d_9_) PC and 15:0–18:1(d_7_)-15:0 TG both contained in the Splash II Lipidomix Mass Spec Internal Standard, 08:0 PE).

### 2.2. Cell Culture and Vitamin B12 or Hydrogen Peroxide Treatment

Human neuroblastoma SH-SY5Y wild-type cells were cultured in a humified incubator at 37 °C and 5% CO_2_ in Dulbecco’s Modified Eagle’s Medium (DMEM), which was free of vitamin B12 and contained 10% FBS (fetal bovine serum; GE Healthcare Life Sciences, Chalfont St. Giles, UK) and 0.1 mM nonessential amino acids. For the incubation of SH-SY5Y cells overexpressing the APP Swedish mutation, 400 µg/mL Hygromycin B was added to the culture medium [22]. When 80% confluence was reached, the FBS content of the medium was reduced to 1% for 16 h, followed by 48 h incubation with 10 nM vitamin B12. The incubation medium was replaced after 24 h. Control cells were treated in parallel with 1‰ water, which was the solvent used to dilute vitamin B12. It is reasonable to assume that a dilution of 1‰ with ddH_2_0 does not affect cell viability and proliferation.

H_2_O_2_ treatment was performed following Zhong et al. (2016) [23]. Four different conditions were distinguished: control, vitamin B12, vitamin B12 in combination with H_2_O_2_, and H_2_O_2_ alone. First, cells were seeded and treated at 70% confluence for twelve hours and followed for 24 h. Control cells were incubated with water as the solvent control, vitamin B12 cells were incubated with 10 nM vitamin B12, and H_2_O_2_ cells were incubated with 50 µM H_2_O_2_, all in DMEM with 1% FCS. For these three conditions, the medium was changed after twelve hours to a medium with the same composition. The vitamin B12 + H_2_O_2_ cells were first pretreated with 10 nM vitamin B12 in DMEM with 1% FCS for twelve hours, followed by a combination of 10 nM vitamin B12 and 50 µM H_2_O_2_ for an additional 24 h. Subsequently, the cells were harvested, and BCA as well as LDH assays were performed as described below. The exact number of biological replicates analyzed in each experiment is given in Supplementary Table S1.

### 2.3. Sample Preparation

After 48 h, the incubated SH-SY5Y cells were harvested at 4 °C. After two washing steps using ice-cold HPLC-grade water, the cells were harvested into 80 µL HPLC-grade water and then homogenized with Minilys (PEQLAB, Erlangen, Germany) using ceramic beads for 30 s at maximum intensity. The bicinchoninic acid (BCA) assay according to Smith et al. was used to determine the sample protein concentration [24]. Homogenates were adjusted to a protein amount of 5 mg/mL in HPLC-grade water. To analyze cell viability, the Cytotoxicity Detection Kit (LDH) from Roche (Basel, Switzerland) was used according to the manufacturer’s protocol. The incubated medium was collected, and the colorimetric assay was used to measure the release of lactate dehydrogenase (LDH) from the cells to quantify cell death and lysis after the vitamin B12 incubations. No significant difference was detected between the cells treated with vitamin B12 and the non-treated control cells. The difference between the vitamin-B12-treated and non-treated cells was less than 0.5%.

### 2.4. Lipid Extraction

A solid/liquid lipid extraction method, previously described in detail in Lauer et al. (2021), was used to detect lipids [25]. A 96-well filter plate (0.45 μm; Merck, Darmstadt, Germany) was attached to a 96-deep-well plate (Fisher Scientific, Schwerte, Germany), and circles of Whatman blotting paper with a diameter of 6 mm were added to each well. Then, both a standard mixture and 10 µL of the prepared sample were added to each Whatman paper. The samples were dried for 45 min under a nitrogen flow (1–2 bar) and then 20 μL of 5% PITC (*v*/*v*) diluted in ethanol/water/pyridine (1:1:1, *v*/*v*/*v*) was added to the samples. Before drying again for 45 min under nitrogen, the samples were incubated for 20 min at room temperature. After drying, lipids were extracted using 300 μL 4.93 mM ammonium acetate in methanol, and the plate was shaken for 30 min at 450 rpm on a plate shaker (IKA, Staufen, Germany). Centrifugation for 2 min at 500× *g* transferred the liquid samples to the 96-well plate, followed by dilution of the samples with 600 μL 5 mM ammonium acetate in methanol/water (97:3, *v*/*v*). After covering the plate with a silicone mat, they were shaken at 450 rpm for 2 min at room temperature and then analyzed by mass spectrometry.

### 2.5. Targeted Shotgun Mass Spectrometry

Lipids that were extracted from a 100 μg sample using the solid/liquid lipid extraction method, as described above, were then subjected to a mass spectrometric analysis, which has been described in detail previously [22,25,26,27]. This shotgun lipidomics process was performed using a 4000-quadrupole linear ion trap (QTrap) equipped with a turbo spray ion source from AB Sciex (Darmstadt, Germany) and coupled to an Agilent HPLC 1200 series autosampler (Santa Clara, CA, USA). The 174 different PCaa, PCae, lyso-PC, carnitine, and TAG species, the 81 different PEaa, PEae, and lyso-PE species, the 45 different PS and lyso-PS species, the 24 different PG species, and the 23 different PI and lyso-PI species were quantified in technical duplicates using the Analyst 1.4.2 software from AB Sciex (Darmstadt, Germany). The exact parameters of the lipid analysis performed in a positive mode were defined in [27].

### 2.6. Gene Expression Analysis

After isolation of total cellular RNA using a TRIzol reagent according to the manufacturer’s guidelines, the quantitative real-time polymerase chain reaction (RT-PCR) experiments were performed. To analyze the purity and concentration of the isolated RNA, its absorbance was measured using a NanoDrop2000 (Thermo Fisher Scientific, Waltham, MA, USA). Subsequently, two micrograms of RNA were reverse transcribed using the High-Capacity cDNA Reverse Transcription Kit as described by the manufacturer to generate complementary DNA (cDNA). Fast SYBR green Master Mix (Applied Biosystems, Foster City, CA, USA) and primers described below on a PikoReal Real-Time PCR System (Thermo Fisher Scientific, Waltham, MA, USA) were used to perform RT-PCR. The most stable housekeeping gene (*RN18S1*; stability value 0.048) out of nineteen examined genes was determined using the NormFinder Algorithm, published in [28], and further used for normalization to exclude differences in RT efficiency. As genes of interest, the expression of the plasmalogens synthesis associated genes *AGPS*, *GNPAT*, *PEDS1*, and *CHPT1*, as well as the expression of the oxidative stress related genes *SOD*, *CAT*, *PRDX2*, *TXNRD1*, and *TXNRD2*, was examined. The used primers are listed in Table 1. Data analysis was performed using the 2-∆∆Cq method. To evaluate how vitamin B12 affects the expression of genes in the presence of H_2_O_2_, H_2_O_2_-treated cells were used as a control and compared to cells treated with H_2_O_2_ and vitamin B12 in combination.

### 2.7. Analysis of β- and γ-Secretase Activity

To examine whether the observed vitamin-B12-mediated changes in the lipid profile of SH-SY5Y cells influence the amyloidogenic processing of APP, the activities of β- and γ-secretases were determined in living SH-SY5Y cells or post-nuclear fractions (PNFs) incubated with lipid extracts derived from vitamin B12 or solvent control treated cells.

SH-SY5Y cells were incubated in 10 cm dishes with vitamin B12 or the solvent control under the conditions described above; afterwards, the lipids were extracted according to Bligh and Dyer [29]. The cells were washed two times with ice-cold HPLC-grade H_2_O and harvested using 180 µL HPLC-grade H_2_O. After mechanical homogenization, the protein content was adjusted to an equal amount, as described above. To extract the lipids, samples were mixed with 1.8 mL of the extraction solution containing CHCl_3_:MeOH (1:2; *v*/*v*) in glass vials and vortexed for 60 min at room temperature. Then, 600 µL CHCl_3_ was added, and the samples were vortexed again for 60 min at room temperature. Finally, 600 µL CHCl_3_ and 600 µL H_2_O were added and the samples were vortexed for another 10 min before they were centrifugated at 5000 rpm for 10 min at room temperature. The lower, lipid-containing phase was transferred into another glass tube and evaporated under nitrogen flow at 30 °C. Thereafter, lipids were dissolved in 100 µL ethanol.

Before the analysis of β- and γ-secretase activity, the SH-SY5Y cells were incubated with lipid extracts from vitamin-B12- or solvent-control-treated cells in a final amount of 3‰ for 48 h in a medium containing 1% FBS.

In the case of the PNF analysis, PNFs were isolated by washing cells in a confluent 10 cm culture dish two times with ice-cold 1xPBS and harvesting them in sucrose buffer (200 mM sucrose, 10 mM Tris/HCl pH 7.4, 1 mM EDTA). Afterwards, the cells were mechanically homogenized and adjusted to an equal protein amount as described before. The PNFs were isolated by sucrose density centrifugation for 10 min at 900× *g* and 4 °C, and 1 mL of the PNFs was transferred into low binding tubes. Incubation with lipid extracts from vitamin-B12- or solvent-control-treated cells was performed in glass vials for 15 min on ice in a final amount of 3‰ corresponding lipid extract.

Afterwards, the activity of β- and γ-secretases was examined using a fluorescence resonance energy transfer (FRET)-based assay as described in [30].

### 2.8. Data and Statistical Analysis

To extract counts per second for each MRM pair, the Analyst 1.4.2. Software from AB Sciex was used. Subsequently, each lipid was normalized to its respective internal lipid class standard and the mean values per duplicate for each lipid/standard ratio per sample were generated. Thereafter, the relative changes compared to the solvent control were calculated and are given in percent in the corresponding bar charts. R (R Core Team 2020; Vienna, Austria; https://www.R-project.org/; accessed on 1 June 2021) was used to perform the statistical analysis, and the two-tailed Student‘s *t*-test was used to calculate the *p*-value for each lipid species shown in the volcano plots. The calculated relative changes compared to the solvent control on the abscissa (represented as the log of the percentage fold change) were plotted logarithmically against the according *p* value on the ordinate. The two vertical lines represent the average standard error of the mean (SEM), which is assessed for each lipid species individually. The binomial test with a 50% likelihood of occurrence of increased lipids was used to determine whether the lipid distribution for lipids beyond the average lipid class SEM is significant. Volcano plots were generated using the R package “EnhancedVolcano” (Kevin Blighe, Sharmila Rana, and Myles Lewis (2020). Version 1.6.0. https://github.com/kevinblighe/EnhancedVolcano; accessed on 1 June 2021).

## 3. Results

In order to examine the potential influence of vitamin B12 on lipids, we treated neuroblastoma cells (SH-SY5Y) with 10 nM vitamin B12 for 48 h (24 + 24 h) and performed a targeted lipidomics approach, including more than 300 different lipid species. Furthermore, to evaluate the anti-oxidative potential of vitamin B12 in regard to the protection of plasmalogen species, we treated SH-SY5Y cells with hydrogen peroxide (H_2_O_2_) in the presence or absence of vitamin B12 and investigated the lipidome as well as the transcription of plasmalogen-synthesis-related and oxidative-stress-related genes.

### 3.1. Phospholipid Species in SH-SY5Y Cells Treated with Vitamin B12

**Figure 1:**
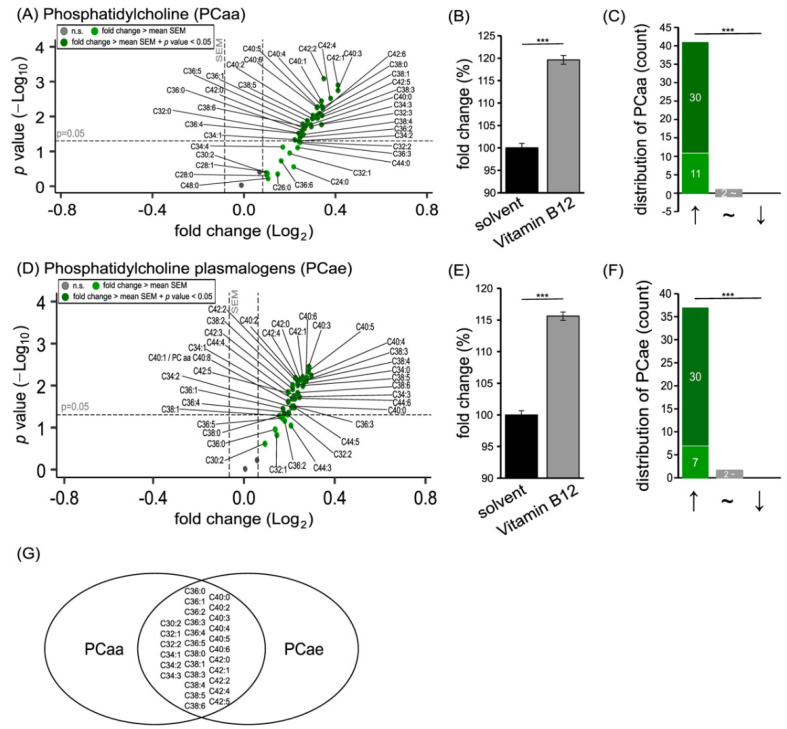
Effect of vitamin B12 treatment on phosphatidylcholine (PCaa) and phosphatidylcholine plasmalogens (PCae) species. SH-SY5Y cells treated with vitamin B12 compared to cells treated with the solvent control (HPLC water). (**A**) In the volcano plot, each PCaa species with its fold change (x-axis) in relation to its *p*-value (y-axis) is shown as a dot. Grey dots represent no significant changes. Light green dots represent a fold change which is greater than the mean standard error of the mean (SEM). Dark green dots represent a fold change which is greater than the mean SEM and, additionally, has a *p*-value smaller than 0.05 (which was defined as the statistical significance level). (**B**) The bar chart shows the relative fold change of all measured PCaa species after vitamin B12 treatment, in comparison to treatment with the solvent control. (**C**) Distribution of PCaa species structured according to the amount of increased, unchanged, or decreased PCaa species. Grey dots represent no significant changes. Light green dots represent a fold change which is greater than the mean standard error of the mean (SEM). Dark green dots represent a fold change which is greater than the mean SEM and, additionally, has a *p*-value smaller than 0.05. (**D**) Volcano plot showing the changes in PCae species due to vitamin B12 treatment. The plot is structured as described in (**A**). (**E**) Representation of the relative fold change of all measured PCae species in a bar chart. (**F**) Distribution of PCae species. Distribution structured as described in (**C**). (**G**) A Venn diagram of PCaa and PCae species is shown. All phospholipids, which underwent alterations greater than the average SEM, are displayed in the overlapping part. Statistical significance was set as *** *p* ≤ 0.001.

**Figure 2:**
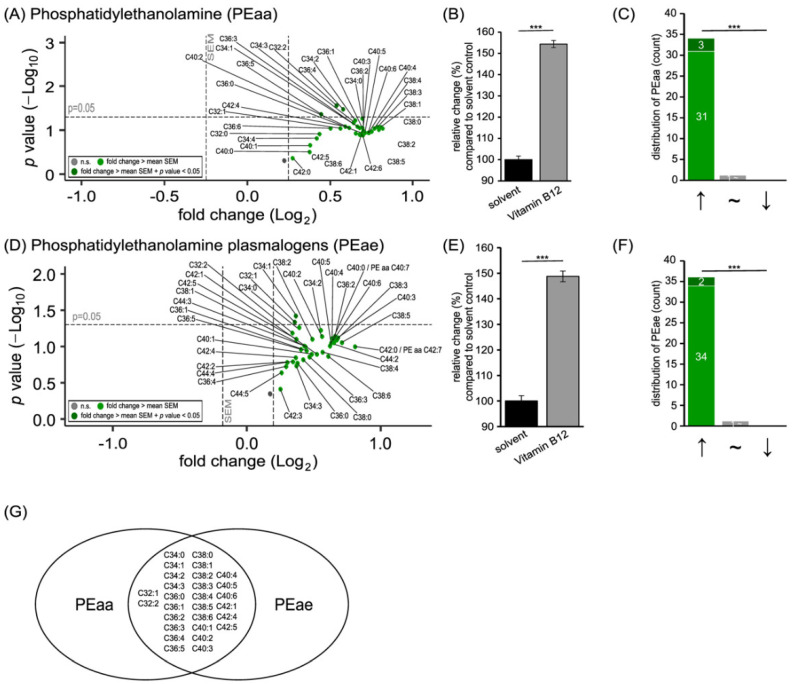
Effect of vitamin B12 treatment on phosphatidylethanolamine (PEaa) and phosphatidylethanolamine plasmalogens (PEae) species. SH-SY5Y cells treated with vitamin B12 compared to cells treated with the solvent control (HPLC water). (**A**) In the volcano plot, each PEaa species with its fold change (x-axis) in relation to its *p*-value (y-axis) is shown as a dot. Volcano plots are structured as in Figure 1. (**B**) The bar chart shows the relative fold change of all measured PEaa species after vitamin B12 treatment, in comparison to treatment with the solvent control. (**C**) Distribution of PEaa species structured according to the amount of increased, unchanged, or decreased PCaa species. Grey dots represent no significant changes. Light green dots represent a fold change which is greater than the mean standard error of the mean (SEM). Dark green dots represent a fold change which is greater than the mean SEM and, additionally, has a *p*-value smaller than 0.05. (**D**) Volcano plot showing the changes in PEae species due to vitamin B12 treatment. (**E**) Representation of the relative fold change of all measured PEae species in a bar chart. (**F**) Distribution of PEae species. Distribution structured as described in (**C**). (**G**) Venn diagram presenting overlapping PEaa and PEae species that underwent changes greater than the average SEM. Statistical significance was set as *** *p* ≤ 0.001.

Phospholipids are the major components of cellular membranes, and phosphatidylcholine (PC) and phosphatidylethanolamine (PE) lipids are most abundant in the plasma membrane fraction of SH-SY5Y cells [31]. Treatment of SH-SY5Y cells with vitamin B12 for 48 hours resulted in significantly elevated levels of PC (see Figure 1) and PE (see Figure 2) species.

The vitamin B12 treatment resulted in 41 out of 43 PCaa species increasing, and a significant elevation to 119.61 ± 0.98% (*p* ≤ 0.001) in total (Figure 1A,B). Among these 41 elevated species, 30 PCaa species were increased significantly due to vitamin B12. This shift in decreased or increased PCaa species was highly significant (*p* ≤ 0.001), as shown in Figure 1C. Similar findings were obtained for PC plasmalogen species, since 37 out of the 39 analyzed PCae species were found to be elevated after treatment with vitamin B12 (Figure 1D). In total, a significant increase to 115.6 ± 0.65% (*p* ≤ 0.001) was observed; this was accompanied by a significant shift from reduced to elevated PCae species (Figure 1E,F). Importantly, both PCaa and PCae species were altered in a similar way. In particular, phospholipids with the fatty acid compositions C30:2, C32:1, C32:2, C34:1, C34:2, C34:3, C36:0, C36:1, C36:2, C36:3, C36:4, C36:5, C38:0; C38:1, C38:3, C38:4, C38:5, C38:6, C40:0, C40:2, C40:3, C40:4, C40:5, C40:6, C42:0, C42:1, C42:2, C42:4, and C42:5 showed the same tendency in PCaa and PCae (Figure 1G). A detailed analysis of the composition of the fatty acids in the examined PCaa and PCae species regarding their saturation state showed that MUFA and PUFA species, especially PUFAs with six double bonds, were significantly increased due to vitamin B12 treatment (Supplementary Figure S1).

Moreover, the treatment of SH-SY5Y cells with vitamin B12 resulted in the levels of PE and PE plasmalogen species increasing significantly, to 154.37 ± 1.69% (*p* ≤ 0.001; PEaa) and 148.77 ± 2.10% (*p* ≤ 0.001; PEae), respectively (Figure 2B,E). Additionally, for these PE species, the shift in decreased or increased species was significant, as 34 out of 35 PEaa species increased and 36 out of 37 PEae species were elevated (*p* ≤ 0.001, respectively) (Figure 2C,F). As shown in the volcano plot, no decreases were detected in the examined PE species, but three PEaa species (C32:2, C36:4, and C36:5) and two PEae species (C32:1 and C34:1) were significantly elevated (Figure 2A,D). Regarding overlapping species, C32:1, C32:2, C34:0, C34:1, C34:2, C34:3, C36:0, C36:1, C36:2, C36:3, C36:4, C36:5, C38:0, C38:1, C38:2, C38:3, C38:4, C38:5, C38:6, C40:1, C40:2, C40:3, C40:4, C40:5, C40:6, C42:1, C42:4, and C42:5 were found to be elevated with an effect strength greater than the SEM (Figure 2G).

**Figure 3:**
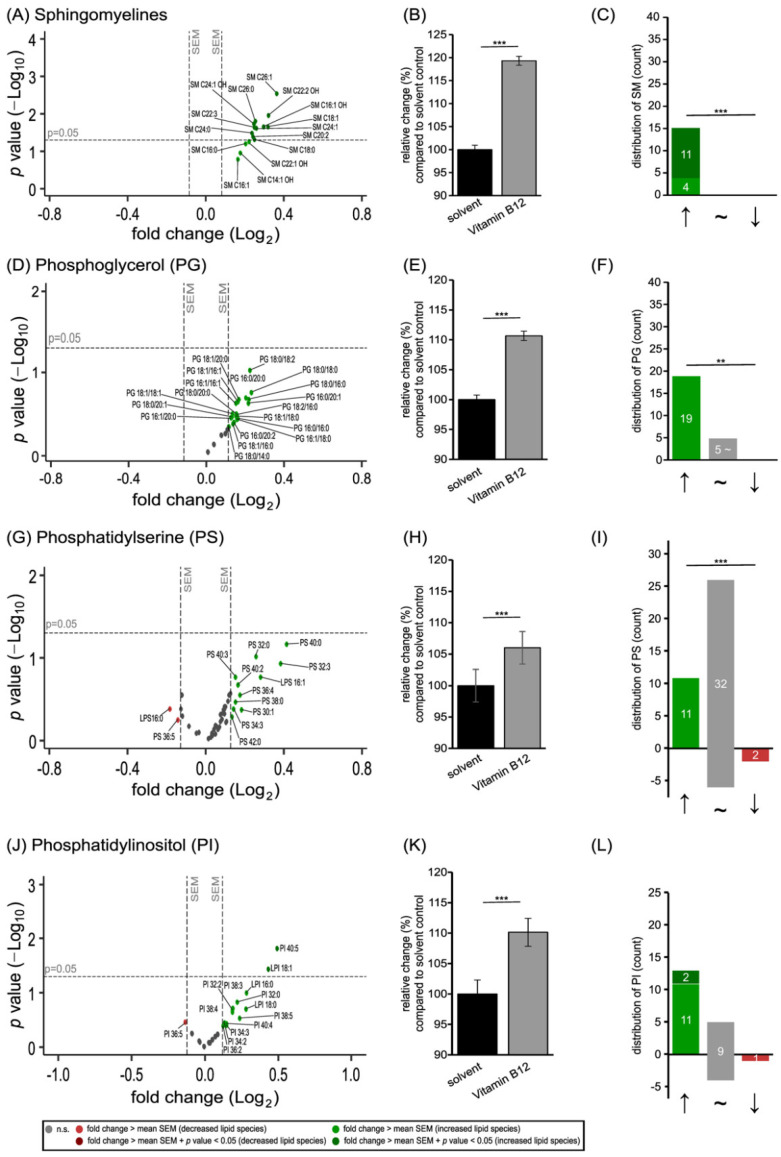
Effect of vitamin B12 treatment on further phospholipids, namely the sphingomyelin (SM, shown in (**A**–**C**)), phosphoglycerol (PG, shown in (**D**–**F**)), phosphatidylserine (PS, shown in (**G**–**I**)), and phosphatidylinositol (PI, shown (**J**–**L**)) species. SH-SY5Y cells treated with vitamin B12 compared to cells treated with the solvent control (HPLC water). In each of the volcano plots (**A**,**D**,**G**,**J**), the particular phospholipid species with its fold change (x-axis) in relation to its *p*-value (y-axis) is shown as a dot. Plots are structured as in Figure 1. (**B**,**E**,**H**,**K**) The bar chart shows the relative fold change of the particular phospholipid species after vitamin B12 treatment, in comparison to treatment with the solvent control. (**C**,**F**,**I**,**L**) Distribution of the particular phospholipid species, structured according to the amount of increased, unchanged, or decreased species. Statistical significance was set as ** *p* ≤ 0.01 and *** *p* ≤ 0.001.

Besides phosphatidylcholines and phosphatidylethanolamines, further phospholipids present in cellular membranes were included in our study. Regarding sphingomyelin (SM), all analyzed species were found to be increased with an effect strength greater than the mean SEM (Figure 3A), resulting in a mean elevation to 119.32 ± 0.97% (*p* ≤ 0.001; Figure 3B). As shown in Figure 3C, 11 out of the 15 SM species were increased significantly, resulting in a significant shift from decreased to increased SM species (*p* ≤ 0.001).

Similar to the SM species, phosphoglycerol (PG) species were also exclusively elevated in SH-SY5Y cells treated with vitamin B12 (Figure 3D). In total, 19 out of the 24 analyzed species were increased with an effect strength greater than the mean SEM (Figure 3F), resulting in a mean increase to 110.66 ± 0.76% (*p* ≤ 0.001; Figure 3E). In line with this, the measured phosphatidylserine (PS) species were increased to 106.00 ± 2.59% (*p* ≤ 0.001; Figure 3H). As represented in Figure 3I, 11 of the 45 PS species were elevated, and two species were reduced without reaching significance. Moreover, 32 PS species remained unaffected by the vitamin B12 treatment according to the definition of effect strengths within the mean SEM (Figure 3G). Regarding phosphatidylinositol (PI) species, an increase to 110.14 ± 2.29% (*p* ≤ 0.001; Figure 3K) was observed after vitamin B12 treatment. As shown in the volcano plot and the distribution diagram (Figure 3J,L), 13 PI species were elevated, with PI 40:5 and Lyso-PI 18:1 reaching significance, and one species (PI 36:5) was reduced; meanwhile, nine species have effect strengths within the mean SEM.

In summary, both the levels of the mainly occurring phospholipids in SH-SY5Y cells (PC, PE and SM), as well as those that occur less frequently, such as PG, PS and PI, were significantly increased in this neuroblastoma cell line due to incubation with vitamin B12.

### 3.2. Further Lipid Species in SH-SY5Y Cells Treated with Vitamin B12

**Figure 4:**
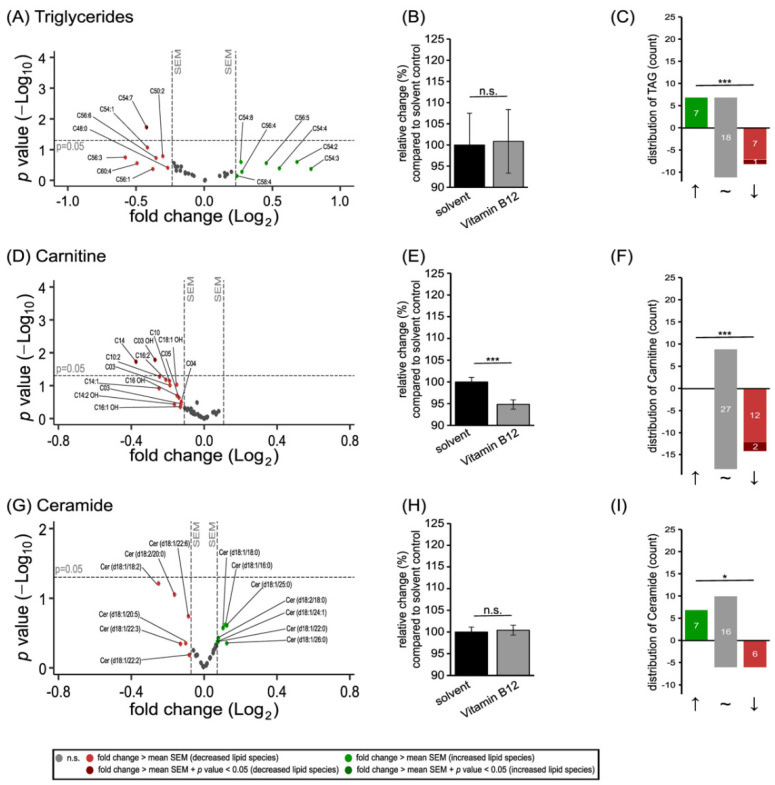
Effect of vitamin B12 treatment on the lipid species triglycerides, carnitines, and ceramides. SH-SY5Y cells treated with vitamin B12 compared to cells treated with the solvent control (HPLC water). In each of the volcano plots (**A**,**D**,**G**) the particular lipid species with its fold change (x-axis) in relation to its *p*-value (y-axis) is shown as a dot. Plots are structured as in Figure 1. (**B**,**E**,**H**) The bar chart shows the relative fold change in the particular lipid species after vitamin B12 treatment, in comparison to treatment with the solvent control. (**C**,**F**,**I**) Distribution of the particular lipid species structured according to the amount of increased, unchanged, or decreased species. Statistical significance was set as * *p* ≤ 0.05, and *** *p* ≤ 0.001 (n.s.: not significant).

In order to examine whether the increase caused by vitamin B12 is specific to phospholipids or is a more universal effect, we investigated the levels of the neutral lipids triglycerides (TAG), the carnitines involved in β-oxidation, and the sphingolipids ceramides in SH-SY5Y cells treated with vitamin B12 (Figure 4).

In contrast to the analyzed phospholipid species, vitamin B12 had no consistent increasing effect on the TAG species included in our study (Figure 4A). Treatment with this water-soluble vitamin resulted in unaffected total levels of TAG (Figure 4B), while seven out of the 33 analyzed TAG species were elevated, seven were reduced, and 18 were unchanged (Figure 4C). The detected carnitine species were significantly reduced to 94.82 ± 1.07% (*p* ≤ 0.001; Figure 4E), as 14 out of the 41 examined species were decreased with an effect strength greater than the mean SEM, whereby C03 OH and C14 were reduced significantly (Figure 4D,F). Regarding ceramides, the results obtained after vitamin B12 treatment were comparable to these of the TAG species, since there was no change in total ceramide levels (Figure 4H). Out of the 29 analyzed ceramide species, seven were increased and six were decreased without reaching statistical significance (Figure 4G,I).

### 3.3. The Anti-Oxidative Properties of Vitamin B12 in the Presence of Hydrogen Peroxide in Relation to Phosphatidylcholine Plasmalogen Species

**Figure 5:**
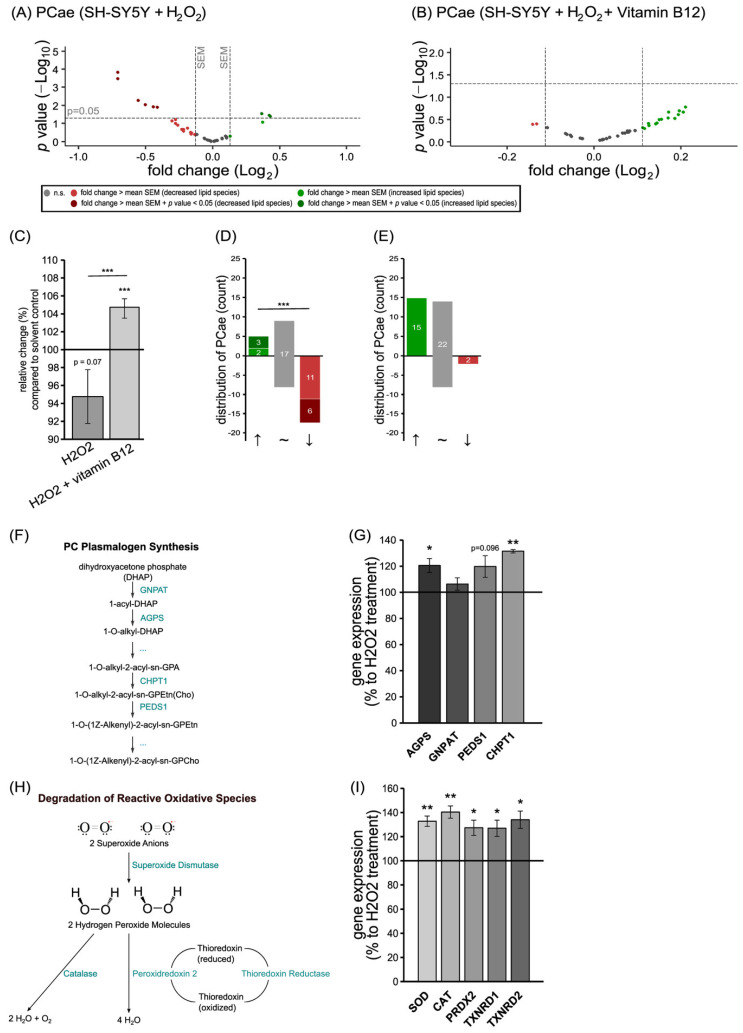
Comparison of the effect of vitamin B12 treatment on phosphatidylcholine plasmalogens (PCae) species in the presence of hydrogen peroxide. SH-SY5Y cells treated with a combination of hydrogen peroxide and vitamin B12, compared to cells treated with hydrogen alone. (**A**,**B**) In each of the volcano plots, each PCae species with its fold change (x-axis) in relation to its *p*-value (y-axis) is shown as a dot. Plots are structured as in Figure 1. (**C**) The bar chart shows the relative fold change of the PCae species after H_2_O_2_ treatment alone or H_2_O_2_ treatment in combination with vitamin B12, in comparison to treatment with the solvent control. (**D**,**E**) Distribution of the PCae species after H_2_O_2_ treatment alone (**D**) or H_2_O_2_ and vitamin B12 treatment (**E**) is shown, structured according to the amount of increased, unchanged, or decreased species. (**F**) Schematic representation of plasmalogen synthesis. (**G**) Expression analysis of genes involved in plasmalogen synthesis using qPCR, comparing combined H_2_O_2_ and vitamin B12 treatment to H_2_O_2_ treatment alone. (**H**) Schematic representation of H_2_O_2_ elimination. (**I**) Expression analysis of genes involved in H_2_O_2_ elimination using qPCR, comparing combined H_2_O_2_ and vitamin B12 treatment to H_2_O_2_ treatment alone. Statistical significance was set as * *p* ≤ 0.05, ** *p* ≤ 0.01 and *** *p* ≤ 0.001.

Plasmalogens are highly sensitive to ROS because of their vinyl ether bond; therefore, we included this lipid species in our experiments, which aimed to evaluate the anti-oxidative properties of vitamin B12. For our investigation, SH-SY5Y cells were incubated with hydrogen peroxide (H_2_O_2_), either alone or with a combination of H_2_O_2_ and vitamin B12; then, the levels of PC plasmalogens (PCae) were analyzed via mass spectrometry. The treatment with H_2_O_2_ alone resulted in a reduction in the total PCae levels to 94.76 ± 3.00% (*p* = 0.074; Figure 5C). As represented in the volcano plot and the distribution diagram (Figure 5A,D), five PCae species were increased, with two of them reaching statistical significance; seventeen PCae species were decreased, with six of them reaching statistical significance. Compared to this, the treatment with H_2_O_2_ in combination with vitamin B12 resulted in a significant shift from reduced PCae species to elevated PCae species ((*p* ≤ 0.001). Due to the combined treatment of vitamin B12 and H_2_O_2_, the levels of PCae were increased to 104.68 ± 1.07% (*p* ≤ 0.001; Figure 5C) compared to the solvent control. Under these combined conditions, fifteen PCae species were found to be increased and two to be decreased (Figure 5B,E).

Using gene expression analysis, we found four genes involved in the plasmalogen synthesis to be increasingly expressed in SH-SY5Y cells treated with the combination of H_2_O_2_ and vitamin B12, compared to cells treated with H_2_O_2_ alone (Figure 5G). The expressions of *glyceronephosphate O-acyltransferase* (*GNPAT*) and *plasmanylethanolamine desaturase 1* (*PEDS1*) tended to be elevated, while expressions of *alkylglycerone phosphate synthase* (*AGPS*) and *choline phosphotransferase 1* (*CHPT1*) were increased significantly due to the presence of vitamin B12 to 120.52 ± 5.35% (*p* = 0.031) and 131.48 ± 1.32% (*p* = 0.002), respectively.

Moreover, under the current experimental conditions, we found vitamin B12 to significantly elevate the expression of genes encoding anti-oxidative enzymes in neuroblastoma cells (Figure 5I). The expression of *superoxide dismutase* (SOD; 132.77 ± 4.35%, *p* = 0.005), *catalase* (CAT; 140.28 ± 5.04%, *p* = 0.004), *peroxiredoxin 2* (PRDX2; 127.32 ± 6.32%, *p* = 0.023), *thioredoxin reductase 1* (TXNRD1; 126.93 ± 6.76%, *p* = 0.028), and *thioredoxin reductase 2* (TXNRD2; 133.98 ± 7.08%, *p* = 0.017) were upregulated significantly.

## 4. Discussion

Alterations in lipid homeostasis are closely linked to the pathogenesis of AD. Several lipids have been shown to alter the proteolytic release of the amyloid-β peptide out of the amyloid precursor protein (APP), Aβ degradation, or the aggregation of Aβ peptides [32,33,34,35,36,37,38]. Beneficial lipids or fatty acids that might potentially reduce the generation of Aβ include, e.g., DHA and plasmalogens [39,40], whereas cholesterol and ceramide elevate Aβ generation [41,42,43,44,45]. Extensive lipid changes have also been found in post mortem AD brain tissue and AD animal models, including changes related to the level of plasmalogens, sphingomyelin (SM), ceramide, cholesterol, and DHA [46,47]. Furthermore, total phospholipids have been found to be reduced in different brain regions in individuals affected by AD [48,49]. Phospholipids account for 20–25% of the adult brain’s dry weight and constitute the backbone of neuronal membranes, including synaptic membranes [50]. Reduced levels of phospholipids therefore result in disease-related deficiencies in synaptic membranes and synapses. In the present study, the neurotropic vitamin B12 was found to significantly increase total phosphatidylcholine (PCaa) levels as well as phosphatidylethanolamine (PEaa) levels. These lipid species have been reported to be decreased in different brain regions and CSF of AD affected individuals [48,51,52,53,54].

In this study, two cellular models were used to investigate the effects of vitamin B12 on lipid homeostasis and the underlying impact to AD. It has to be emphasized, that cellular models are widely used to elucidate the molecular mechanism of action of therapeutics, which might have a beneficial effect for AD, but further studies are needed to verify these potential benefits as a therapeutic in vivo, utilizing animal models and clinical studies. Therefore, our results aimed to point out that vitamin B12 plays a crucial role in lipid homeostasis and oxidative stress and that these changes are able to modify the amyloidogenic secretases involved in AD in neuroblastoma cells. However, these data are based on cell culture findings and should be interpreted carefully.

First, we have used the SH-SY5Y WT cells, which is a neuroblastoma cell line with neuronal properties, widely used to investigate the molecular mechanisms leading to AD in the context of sporadic AD, which is not caused by mutations. This model is in particular used as an in vitro model in AD research for drug development [55].

Beside the sporadic form, AD can be caused by so called familial AD mutations, mostly found in *APP*, *PS1* or *PS2*. To verify, if our findings are also obtained under these conditions, we have used the APPswedish (APPswe) overexpressing SH-SY5Y cell line. These data are presented in the Supplementary Figures S3 and S4. The APPswe mutation is a double mutation near the β-secretase cleavage side of APP (wild-type: KM; APPswe: NL). The mutated APP is primarily processed by β-secretase and therefore causes elevated level of Aβ and the transcriptional regulator AICD via the amyloidogenic processing pathway [56,57,58,59]. SH-SY5Y cells expressing the APPswe mutation are a commonly accepted cellular AD-model and frequently used in AD research, for example in the context of oxidative stress [60,61,62,63,64,65,66,67]. Importantly, this cell line has been characterized for its suitability to be used as a familial AD model, for details please see [61]. In summary, it has to be pointed out that several FAD models exist [55], and each model has its individual benefits and has to be carefully selected in respect to its experimental questioning. As a proof of concept, we decided to analyze potential lipid changes in this FAD model compared to WT cells, specifically in respect to plasmalogens and compared these data with known and consistent results obtained from human AD post mortem brains.

SH-SY5Y cells overexpressing the APPswe mutation revealed a significant decrease in plasmalogen levels. As shown in Figure S3, all 39 detected PCae species were reduced in SH-SY5Y APPswe cells compared to wild-type cells, incubated with the solvent control respectively (Figure S3A). In total, PCae level were decreased in APPswe cells in comparison to SH-SY5Y WT (Figure S3B), indicating that SH-SY5Y cells stably expressing the APPswe mutation are suitable to address the effect of vitamin B12 on AD. These results are in line with literature, consistently showing a decrease in plasmalogen levels in different brain regions or CSFs in AD patients [26,48,68,69,70,71,72,73]. Furthermore, reduced plasmalogen levels can be found in AD mouse models [74,75,76].

As the effect of vitamin B12 on plasmalogen levels is one of the most pronounced effects of vitamin B12, we decided to use this APPswe cell line as a familial AD model system. Notably, after vitamin B12 treatment, PCaa and PCae levels were not only elevated in SH-SY5Y WT cells, but also in SH-SY5Y APPswe cells, illustrating that vitamin B12 is also able to affect lipid homeostasis in a cellular AD model (Figure S4). In summary, these findings suggest that vitamin B12 might be beneficial in both the sporadic and the familial forms of AD. In SH-SY5Y WT cells, 14 out of the 41 carnitine species were decreased with an effect strength greater than the mean SEM due to vitamin B12 (Figure 4D,F). Additionally, comparable lipid-reducing effects of vitamin B12 were found for APPswe cells. The incubation with vitamin B12 resulted in 16 reduced species with an effect strength greater than the mean SEM, and three significantly reduced carnitine species (Figure S4E,F). Interestingly, vitamin B12 injections have been reported to increase levels of plasmalogens and further phospholipids in elderly individuals with vitamin B12 deficiency [77], which was also found in both cell lines after vitamin B12 treatment; this further indicates that the altered lipid changes are not limited to the cellular model used in our study. Further studies are needed to confirm these effects in vivo.

Potential explanations for the observed lipid changes and potential beneficial properties of vitamin B12 in respect to AD are given in the Supplementary Discussion as further background information. However, these explanations are based on literature and should not be overinterpreted without further experimental proof [78,79,80,81,82,83,84,85,86,87,88,89,90,91,92,93,94,95,96,97,98,99,100,101,102,103,104,105,106,107,108,109,110,111,112,113,114,115,116,117,118,119,120].

In the present study, vitamin B12 treatment of SH-SY5Y cells led to a significant increase in both phosphatidylcholine plasmalogens and phosphatidylethanolamine plasmalogens in wild-type neuroblastoma cells and in a cellular AD-model expressing the Swedish mutation of APP. In this context, it should be mentioned that low serum vitamin B12 levels and reduced plasmalogen levels are not only reported for AD but also for Parkinson’s disease [121,122], meaning that our findings could also be considered in relation to other neurodegenerative disorders, especially considering that vitamin B12 is also reduced in vascular dementia [123].

Furthermore, vitamin B12 protected plasmalogens from oxidation, as we found a reduction in plasmalogens in the presence of hydrogen peroxide (H_2_O_2_), which could be reverted by co-treatment with vitamin B12. The change in the reduced plasmalogen species in the presence of H_2_O_2_, versus increased plasmalogen species in the presence of H_2_O_2_ and vitamin B12, was highly significant. In this context, it should be mentioned that we preferred to use H_2_O_2_ to induce oxidative stress in our cell culture system as H_2_O_2_, one of the most important ROS species, is present in higher levels in the brains of AD-affected individuals compared to healthy control brains, and plays an important role in the progression of the disease [124,125]. The close association of H_2_O_2_ with AD is further supported by the fact that Aβ peptides increase H_2_O_2_ formation and H_2_O_2_-forming enzyme activities [126]. A dose-dependent protective effect of vitamin B12 against H_2_O_2_-induced apoptosis in SH-SY5Y cells was recently reported. The significant protective effects of vitamin B12 were already apparent at 2 µM vitamin B12 [23], which promoted cell survival. These antioxidative neuroprotective properties, whereby vitamin B12 protects cells from cytotoxicity, was also reported for Aβ-induced oxidative damage. PC12 cells that were chronically exposed to Aβ25-35 peptides to establish an AD cell model for Aβ-induced toxicity revealed an increase in oxygen radicals and nitric oxide. The co-treatment of PC12 cells exposed to Aβ25-35 peptides with methyl-vitamin B12 improved cell viability by decreasing the percentage of apoptotic cells in the presence of vitamin B12 compared to controls [127]. The protective effect of vitamin B12 in respect to cell viability was also shown for SH-SY5Y cells exposed to 70-hour-aged Aβ42 amyloids [128]. In the presence of Aβ42 aggregates, the cell viability of SH-SY5Y cells was decreased to 32%, whereas in the presence of 25 and 50 µM vitamin B12, cell viability was elevated to 74% and 83%, respectively.

**Figure 6:**
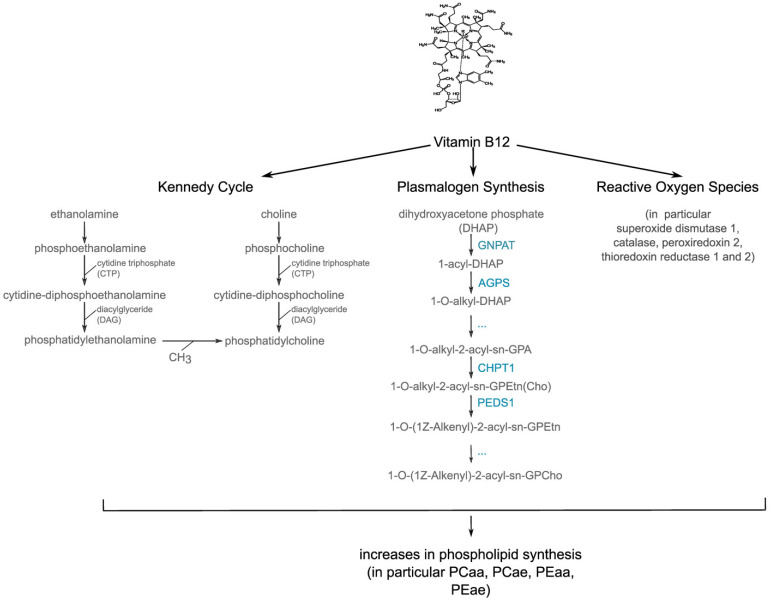
Schematic summary of the link between vitamin B12, the Kennedy cycle, plasmalogen synthesis, and reactive oxygen species (ROS).

The potential mechanisms underlying the vitamin-B12-induced protection of plasmalogens from being oxidized could be attributed to the increased expression of genes that encode anti-oxidative enzymes. The co-treatment of SH-SY5Y cells with H_2_O_2_ and vitamin B12 leads to a significant increase in the expression of *superoxide dismutase* (SOD), *catalase* (CAT), *peroxiredoxin 2* (PRDX1), *thioredoxin reductase 1*, and *thioredoxin reductase 2* (TXNRD1 and 2), compared to SH-SY5Y cells treated with H_2_O_2_ alone (Figure 5). These enzymes are important for the degradation of reactive oxidative species (Figure 6). Similarly to our findings regarding vitamin B12, a recent study dealing with the antioxidative function of resveratrol in an AD mouse model found increased expression and activity of *SOD* and *catalase* in resveratrol-treated mice compared to control mice [129]. Interestingly, the activity of these enzymes has been reported to be reduced in human post mortem frontal cortexes from individuals characterized either with MCI or mild/moderate AD, as well as late-stage AD [130,131]. Furthermore, *SOD* activity was significantly decreased in the AD frontal and AD temporal cortex, while *CAT* activity was significantly decreased in the AD temporal cortex compared to age-matched controls [132]. Reduced *SOD* activity has also been reported for the plasma and red blood cells of elderly individuals with MCI and AD [133].

Beside the observed significantly elevated expression of genes involved in ROS degradation in the presence of H_2_O_2_ and vitamin B12, we found that vitamin B12 increased the expression of genes involved in plasmalogen synthesis (Figure 5). Exposure of SH-SY5Y cells to H_2_O_2_ and vitamin B12 significantly elevated the expression of the *alkylglycerone phosphate synthase* (AGPS) and *choline phosphotransferase 1* (CHPT1), compared to cells treated with H_2_O_2_ alone. *Glyceronephosphate O-acyltransferase* (*GNPAT*) and *plasmanylethanolamine desaturase* 1 (PEDS1) tended to be elevated in the presence of H_2_O_2_. These results indicate that vitamin B12 increases plasmalogen levels by protecting them from being oxidized, and by elevating plasmalogen de novo synthesis in cells exposed to oxidative stress. Vitamin B12 therefore represents a potential micronutrient to compensate reduced plasmalogen levels, which are commonly seen in brains affected by AD. Moreover, a recent study also reported that vitamin B12 reduces TDP-43 (TAR DNA-binding protein 43) toxicity in SH-SY5Y cells by reducing oxidative stress and mitochondrial dysfunction [134]. Cytoplasmic aggregation of TDP-43 represents a pathological hallmark of many neurodegenerative diseases, including AD, amyotrophic lateral sclerosis (ALS), frontotemporal dementia, and limbic predominant age-related TDP-43 encephalopathy, indicating that vitamin B12 is a possibly beneficial therapeutical micronutrient for several neurodegenerative diseases.

This potential therapeutic use for vitamin B12 is further suggested by studies dealing with the effect of vitamin B12 on the amyloid- and tau-pathology of AD. It has been shown that vitamin B12 reduces amyloid pathology by different mechanisms. These include the decreased gene expression of BACE1 and PS1 [135,136,137,138], two important enzymes for the release of Aβ peptides out of APP [139,140,141,142]; decreased cholesterol de novo synthesis [143]; and the inhibition of Aβ fibrillization and aggregation [128,144]. The protective effect of vitamin B12 in respect to tau hyperphosphorylation and tau aggregation is reported to be caused by an elevation in the activity of the phospholipase A2 [145], an inhibition of kinases involved in tau phosphorylation e.g., GSK-3β [146], and by the direct binding of vitamin B12 to tau proteins [147]. In line with the obtained and discussed positive properties of vitamin B12 in respect to the lipid alterations found in AD, several clinical randomized controlled trials with patients affected by mild cognitive impairment or AD have already revealed the beneficial effects of vitamin B12 when used alone or in combination with vitamin B6 or folic acid [115]. These results further underline the therapeutic potential of vitamin B12 to treat or prevent AD.

In summary, our results show a crucial link between vitamin B12 and lipid homeostasis and oxidative stress. Vitamin-B12-induced changes are able to have beneficial effects on the amyloidogenic processing of APP, further indicating its potential useful properties in respect to AD. Even though vitamin B12 supplementation has only marginal or no known side effects under physiological doses, further studies are needed before an unambiguous recommendation can be made.

## Figures and Tables

**Table 1:** **Forward and reverse primers used for gene expression analysis of plasmalogen synthesis or oxidative-stress-related genes.** AGPS: alkylglycerone phosphate synthase; GNPAT: glyceronephosphate O-acyltransferase; PEDS1: plasmanylethanolamine desaturase 1; CHPT1: choline phosphotransferase 1; SOD: superoxide dismutase; CAT: catalase; PRDX2: peroxiredoxin 2; TXNRD1: thioredoxin reductase 1; TXNRD2: thioredoxin reductase 2; RN18S1: 18S ribosomal RNA 1; HKG: housekeeping gene.

Gene Name	Forward Primer (5′-3′)	Reverse Primer (5′-3′)
*AGPS*	ACCAGATTCCCTGGAGTTCA	GAACCACCAGGTCCTCGATA
*GNPAT*	TACAACTGGGTTCTGAAAGCC	CAGCTGCCAAAGATCGAAGT
*PEDS1*	ACCATCGCATCCACCACGTC	AGGCGTCGCCAGAAGCCTAT
*CHPT1*	TCCAGTTCTTGGATTTCTAGGTGGAGT	ACACTGGTGCCTGCTATAGTGGA
*SOD*	CAGCAGGCTGTACCAGTGC	ACATTGCCCAAGTCTCCAAC
*CAT*	ATTCGATCTCACCAAGGTTTG	CTTGGGTCGAAGGCTATCTG
*PRDX2*	CACCTGGCTTGGATCAACA	GCCGTAATCCTCAGACAAGC
*TXNRD1*	ACACAAAGCTTCAGCATGTCA	CAATTCCGAGAGCGTTCC
*TXNRD2*	GCATGACTGGAGGAAGATGG	AAACCGTGTGCTCGTCAAC
*RN18S1* (HKG)	GGAGTATGGTTGCAAAGCTGA	ATCTGTCAATCCTGTCCGTGT

## Data Availability

Not applicable.

## Supplementary Materials

The following supporting information can be downloaded at: https://www.mdpi.com/article/10.3390/cells11162574/s1, Figure S1: Effects of Vitamin B12 on the saturation state of PCaa and PCae species; Figure S2: Effects of vitamin B12 on β- and γ-secretase in SH-SY5Y WT and SH-SY5Y APPswe cells; Figure S3: Effect of vitamin B12 treatment on phosphatidylcholine plasmalogens (PCae); Figure S4: Effect of vitamin B12 treatment on phosphatidylcholine (PCaa), phosphatidylcholine plasmalogens (PCae) and carnitine species; Table S1: Number of biological replicates analyzed in each experiment; Supplementary Discussion; Table S2: Lipid changes of the most abundant phospholipids in neuronal membranes due to vitamin B12 treatment in SH-SY5Y WT cells and their link to AD.
