# Supplementary material for: Vitamin B12 Attenuates Changes in Phospholipid Levels Related to Oxidative Stress in SH-SY5Y Cells
Source: Cells. 2022 Aug 18;11(16):2574. doi: 10.3390/cells11162574 (PMC9406929; doi:10.3390/cells11162574)
Supplement: Supplementary file 1 [file cells-11-02574-s001.zip › cells-1556674-supplementary.pdf]

Supplementary Materials

Vitamin B12 attenuates changes in phospholipid levels related to oxidative stress (in SH-SY5Y cells)

Elena Leoni Theiss [1, †], Lea Victoria Griebsch [1, †], Anna Andrea Lauer [1], Daniel Janitschke [1], Vincent Konrad Johannes Erhardt [1], Elodie Christiane Haas [1], Konstantin Nicolas Kuppler [1], Juliane Radermacher [1], Oliver Walzer [1], Dorothea Portius [2], Heike Sabine Grimm [1], Tobias Hartmann [1,3] and Marcus Otto Walter Grimm [1,3,4,*]

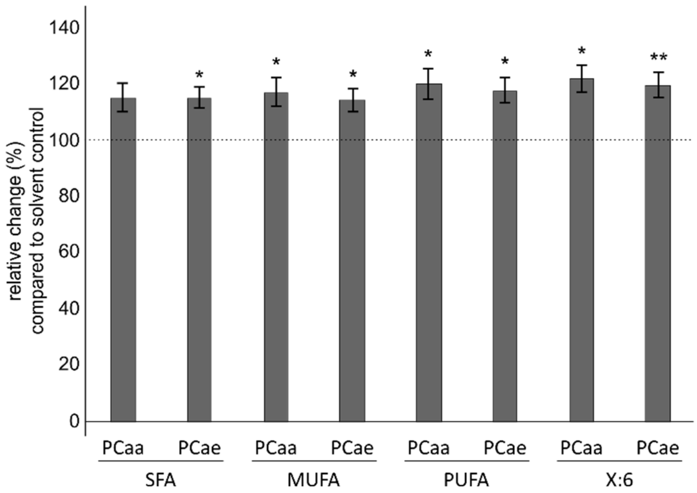

Supplementary Figure S1. Effects of Vitamin B12 on the saturation state of PCaa and PCae species. The relative changes of saturated fatty acid- (SFA), monounsaturated fatty acid- (MUFA), polyunsaturated fatty acid- (PUFA) and PCaa / PCae species containing fatty acids with six (X:6) double bonds in SH-SY5Y cells treated with Vitamin B12 compared to cells incubated with the solvent control. Statistical significance was set as * $p \leq 0.05$ and ** $p \leq 0.01$.

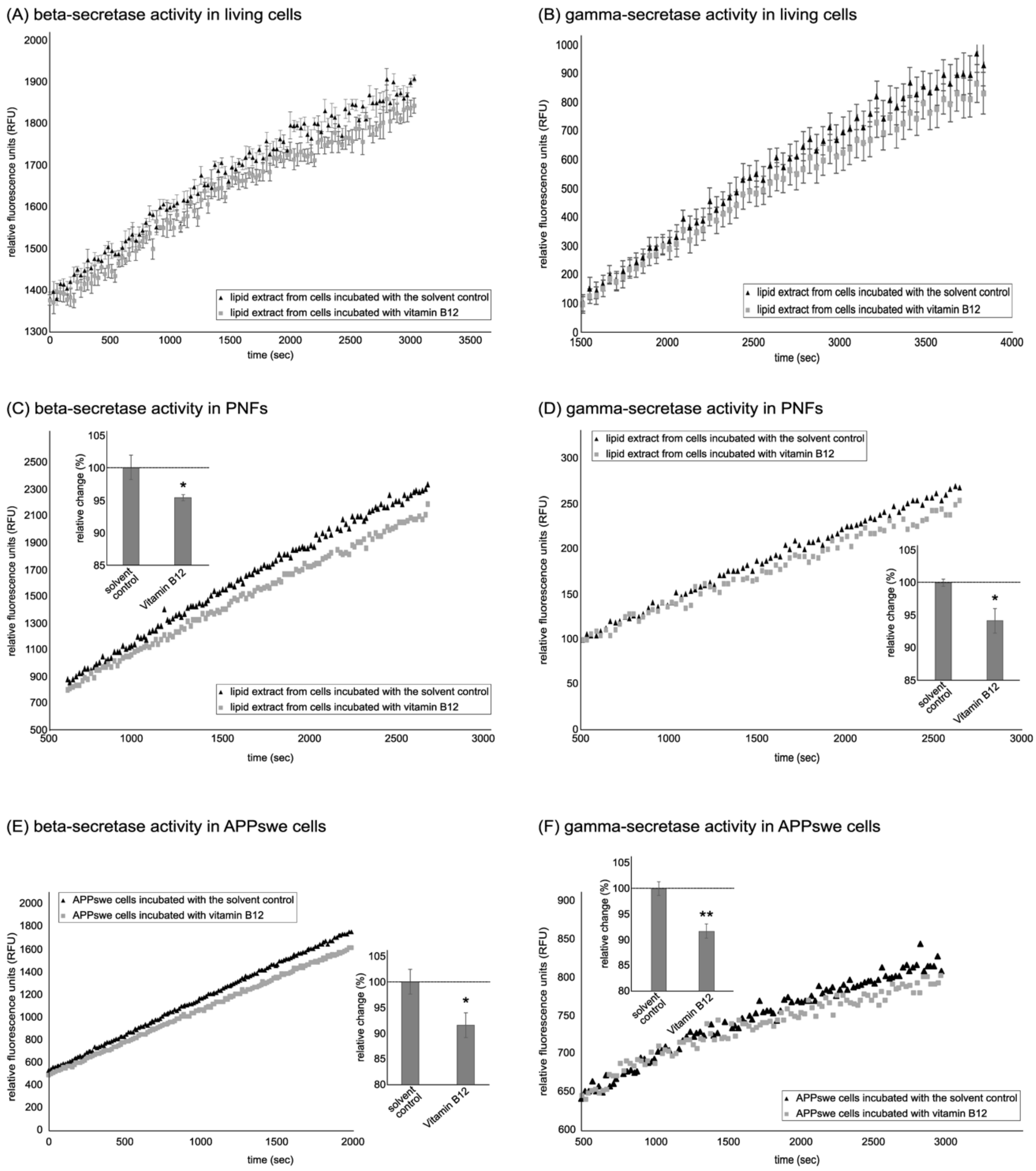

Supplementary Figure S2. Effects of vitamin B12 on β- and γ-secretase in SH-SY5Y WT and SH-SY5Y APPswe cells. Lipid extracts from cells incubated with the solvent control or vitamin B12 were incubated for 48 hours on SH-SY5Y WT cells (**A-B**) or for 15 min at 4 °C on post-nuclear fractions (PNFs; **C-D**) of SH-SY5Y cells and the activity of β-secretase and γ-secretase were determined using a fluorescence-resonance-energy-transfer (FRET) assay. β-secretase and γ-secretase activities measured in homogenates of SH-SY5Y APPswe cells incubated for 48 hours with the solvent control or vitamin B12 (**E-F**). One representative kinetic for each secretase is shown. Statistical significance was set as * $p \leq 0.05$ and ** $p \leq 0.01$.

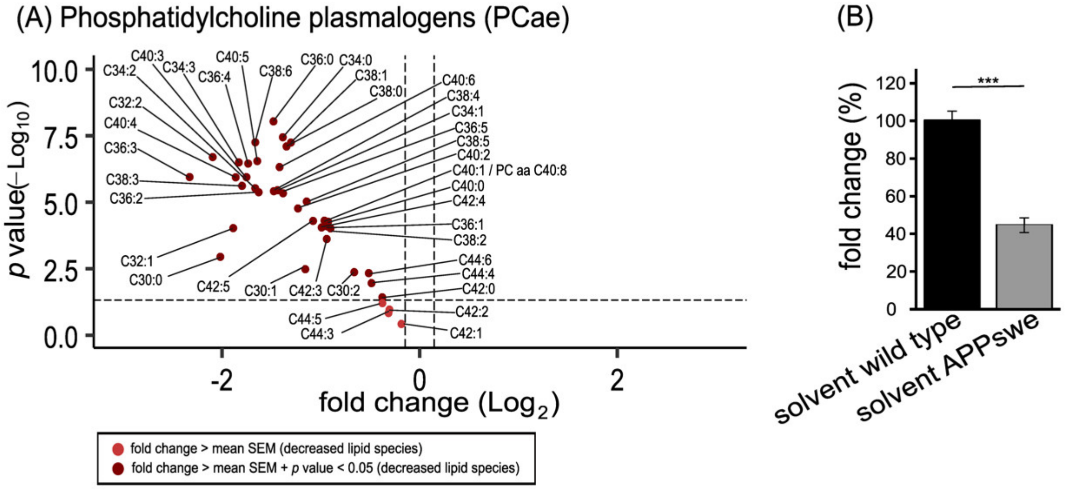

Supplementary Figure S3. Effect of vitamin B12 treatment on phosphatidylcholine plasmalogens (PCae). SH-SY5Y APPswedish cells treated with the solvent control (HPLC-water) compared to SH-SY5Y cells treated with the solvent control (HPLC-water). (**A**) In the volcano plot each PCae species with its fold change (x-axis) in relation to its p-value (y-axis) is shown as a dot. Light red dots represent a fold change, which is smaller than the mean standard error of the mean (SEM). Dark red dots represent a fold change, which is smaller than the mean SEM and additionally have a p-value smaller than 0.05 (which was defined as statistical significance level). (**B**) In the bar chart the relative change of all measured PCae species after solvent control treatment in SH-SY5Y APPswedish cells compared to the solvent control treatment of SH-SY5Y wildtype cells is shown. Statistical significance was set as *** $p \leq 0.001$.

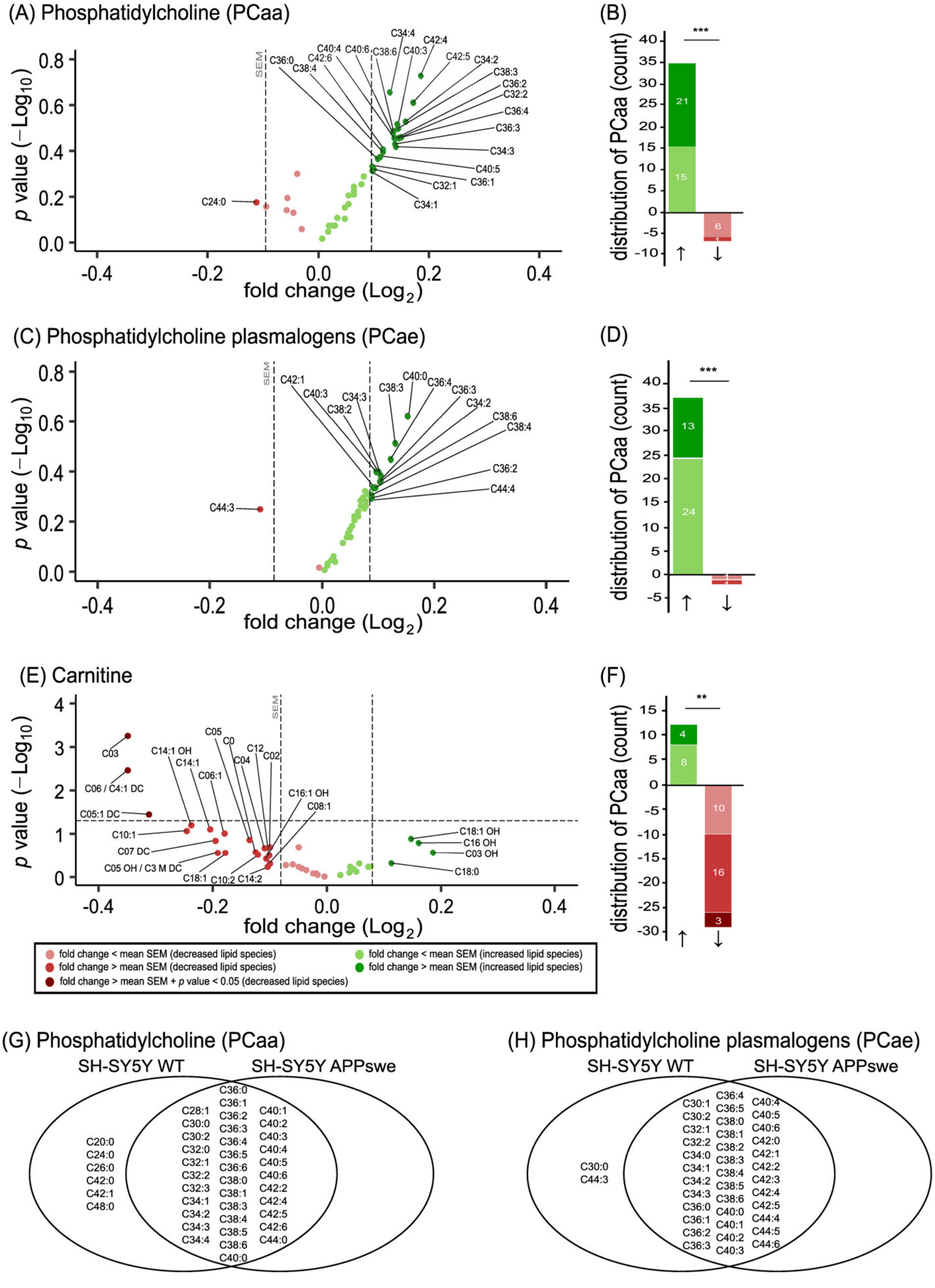

Supplementary Figure S4. Effect of vitamin B12 treatment on phosphatidylcholine (PCaa), phosphatidylcholine plasmalogens (PCae) and carnitine species. SH-SY5Y APPswedish cells treated with vitamin B12 compared to cells treated with the solvent control (HPLC-water). In each of the volcano plots (**A,C,E**) the particular lipid species with its fold change (x-axis) in relation to its p-value (y-axis) is shown as a dot. Very light green and very light red spots represent no significant changes. Light green dots

represent a fold change, which is greater than the mean standard error of the mean (SEM). Light red dots represent a fold change, which is smaller than the mean standard error of the mean (SEM). Dark red dots represent a fold change, which is smaller than the mean SEM and additionally have a p-value smaller than 0.05. (**B,D,F**) Distribution of the particular lipid species structured in the amount increased or decreased species. Statistical significance was set as ** $p \leq 0.01$ and *** $p \leq 0.001$. (**G**) A Venn diagram of PCaa wild type and PCaa APPswedish species is shown. All altered up-regulated phospholipids are displayed in the overlapping part. (**H**) A Venn diagram of PCae wild type and PCae APPswedish species is shown. All altered up-regulated phospholipids are displayed in the overlapping part.

Supplementary Table S1: Number of biological replicates analyzed in each experiment.

Figure	Biological replicates
Figure 1	solvent control: n=23; Vitamin B12 treatment: n=26
Figure 2	solvent control: n=23; Vitamin B12 treatment: n=26
Figure 3	solvent control: n=23; Vitamin B12 treatment: n=25
Figure 4	solvent control: n=23; Vitamin B12 treatment: n=26
Figure 5	solvent control: n=8; H_2O_2 treatment: n=8<br>combined treatment with H_2O_2 and Vitamin B12: n=9

Supplementary discussion:

The observed vitamin B12-induced increase in total phosphatidylcholine might be attributed to the important role of vitamin B12 in the homocysteine/methionine cycle. Vitamin B12 is an essential cofactor for the 5-methyltetrahydrofolic acid dependent methionine synthase, which catalyzes the synthesis of methionine from homocysteine [78,79]. Methionine is then converted to s-adenosylmethionine (SAM), a very important methyl-group donor to a variety of genomic and non-genomic substrates including the methylation-dependent generation of phosphatidylcholines, the most abundant phospholipids in neuronal membranes, in the Kennedy pathway (Figure 8). In this context it has to be mentioned that a nutrient combination containing uridine, DHA and choline, which are important for PC synthesis in the Kennedy cycle, increases neurite outgrowth and synaptogenesis [80]. Furthermore, a recent cell culture study found that supplementation of Fortasyn connect (FC), containing beside other supplements uridine, DHA, choline and vitamin B12, to primary neuron-astrocyte co cultures resulted in an elevated number of neurons without affecting astrocyte number. FC also improved memory performances in early AD patients [81] emphasizing the effect of FC on synaptogenesis. Importantly, the Kennedy pathway also generates the phosphatide phosphatidylethanolamine, that level has also been found to be increased in SH-SY5Y cells supplemented with vitamin B12. The described beneficial property of vitamin B12 in respect to phospholipid synthesis is further underlined by a slight but significant increase in phosphoglycerol, phosphatidylserine and phosphatidylinositol in our cell culture system. Notably, phosphatidylinositol level was found to be decreased in different brain regions of AD affected individuals [53,82,83], but unaffected in CSF [84]. In contrast, phosphatidylserine levels were reported to be decreased in CSF of patients suffering from MCI or AD [52].

In our study, vitamin B12 treatment also resulted in a significant increase in sphingomyelin, an important membrane lipid, especially enriched in the central nervous system. Although the results of

studies analyzing the content of SM in AD-affected brains are inhomogeneous [82,85–87], plasma sphingomyelins are reported to predict slower progression among AD patients [88] and an altered sphingolipid metabolism is closely linked to AD [89]. Furthermore, soluble Aβ peptides have been shown to increase the activity of neutral and acid sphingomyelinases [90,91], thus downregulating SM level and increasing ceramide level, known to be elevated in different brain regions and CSF of AD-patients [85,92–96] and to increase Aβ production [43–45]. As we found unchanged levels of the sphingolipid ceramide in presence of vitamin B12, the observed increase in SM might be attributed to the effect of vitamin B12 on total phosphatidylcholine level in the Kennedy cycle as SM is the only sphingolipid being simultaneously a phospholipid formed from phosphatidylcholines. Importantly, it has been reported that myelin impairment may play an important role in AD pathology and that myelin pathology might even precede Aβ and tau pathologies in AD [97,98]. Vitamin B12 affects the formation of myelin by affecting DNA synthesis of myelin-producing oligodendrocytes [99–101]. SM is especially enriched in the myelin sheets surrounding axons of many nerves, possibly indicating that the vitamin B12-induced elevation in SM might also protect from myelin damage.

Plasmalogens, major constituents of neuronal membranes, are discussed to be closely related to AD [102]. Plasmalogens are glycerophospholipids characterized by a vinyl ether bond at the sn-1 position of the glycerol backbone, which makes plasmalogens more susceptible to oxidative stress than the corresponding ester-bonded glycerophospholipid. In human brain, plasmalogens represent almost 20% of total glycerophospholipids. Decreased levels of plasmalogens have been commonly found in post mortem AD brain samples as well as in CSF [26,48,52,72,73,82,103,104], plasma, serum and red blood cells of AD patients [105–107]. This decrease in plasmalogens was observed in brain regions mostly affected by AD, hippocampus, temporal and frontal cortex [72,103]. This loss in plasmalogens in AD brain might be related to oxidative stress, leading to the degradation of plasmalogens by ROS [108]. However, as plasmalogens themselves act as antioxidants protecting other lipids and lipoproteins from oxidation [109,110], the diminished plasmalogen levels may further promote ongoing oxidative damage in AD [108]. Potential molecular mechanisms that are linked to beneficial properties of plasmalogens are discussed to be related to a decreased Aβ release out of APP as well as a reduction in Aβ aggregation by plasmalogens [39,102,111]. Furthermore, the APP intracellular domain that is generated by the Aβ-producing amyloidogenic APP processing pathway, has been shown to decrease the expression of the alkylglycerone phosphate synthase (AGPS), a rate limiting enzyme in plasmalogen synthesis [112]. To evaluate if the observed vitamin B12-induced changes in the lipid profile are able to affect AD-related processes like amyloidogenic cleavage of APP, we performed a lipid extraction after incubating the SH-SY5Y cells with either vitamin B12 or the solvent control and incubated these lipid extracts on cells before determining the activity of β- and γ-secretases. In line with our previous findings, the activities of β- and γ-secretase were slightly but, in case of the β-secretase, significantly reduced (β-secretase, p = 0.009; γ-secretase, p = 0.081; see Supplemental Figure S2A,B). Similar results were obtained when the lipid extracts were incubated on post-nuclear fractions (PNF) since β- and γ-secretase were also slightly but significantly reduced (β-secretase to 95.3 ± 0.5% (p = 0.041) and γ-secretase to 94.2 ± 1.9% (p = 0.049); Supplemental Figure S2C,D). Moreover, the incubation of a cellular AD-model (SH-SY5Y APPswe cells) with vitamin B12 resulted in significantly decreased activities of β-secretase to 91.5 ± 2.4% (p = 0.048) as well as γ-secretase to 91.7 ± 1.4% (p = 0.003) measured in cell homogenates (Supplemental Figure S2E,F).

Explanations of the vitamin B12-mediated lipid changes and their relevance in relation to AD are summarized in Table 2.

Table S2. Lipid changes of the most abundant phospholipids in neuronal membranes due to vitamin B12 treatment in SH-SY5Y WT cells and their link to AD.

Lipid Class	Vitamin B12	Lipid Changes Found in AD-Affected Individuals and Examples of Proposed AD-Related Mechanisms
Plasmalogens (PCae, PEae)	↑	Plasmalogens have been found to be decreased in AD post mortem brains [26,48,72,73,82,103] as well as in CSF, plasma, serum and red blood cells of AD-affected individuals [52,105–107]. This decrease in plasmalogens is discussed to be caused by an increase in plasmalogen degradation by elevated oxidative stress found in AD as well as by a reduction in plasmalogen de

		novo synthesis caused by a general decrease in peroxisomal function [8,108]. The potential protective properties of plasmalogens in respect to AD pathogenesis are reported to be attributed to a diminished amyloidogenic APP processing as well as a reduction in Aβ aggregation [39,102,111]. Furthermore, the intracellular domain of APP has been found to decrease the expression of the alkylglycerone phosphate synthase, an important enzyme in plasmalogen synthesis [112]. A plasmalogen-induced reduction in transcriptionally active AICD would in return elevate plasmalogen synthesis.
Phosphatidylcholine (PCaa)	↑	Several studies reported reduced PC level in AD post mortem brains as well as in CSF of AD patients [48,51,52]. PC are the most abundant phospholipids in neuronal membranes and therefore important for synaptogenesis. Loss of synapses correlates to cognitive deficits in AD and numerous publications report post mortem synapse loss in AD patients summarized in [113]. The vitamin B12-induced increase in PC can be attributed to the important function of vitamin B12 as cofactor for the methionine synthase converting homocysteine, a known risk factor for AD [114], to methionine, further converted to s-adenosylmethionine, important for the methylation of phosphatidylethanolamine generating PC [115].
Phosphatidylethanolamine (PEaa)	↑	PE level are reported to be reduced in AD post mortem brains and CSF of AD affected patients [51–54]. This decrease in PE (as well as PC) is discussed to be due to a higher phospholipid turnover in AD brain tissue [51]. PE synthesis strongly depends on the Kennedy pathway, closely linked to the vitamin B12 dependent homocysteine/methionine cycle.
Sphingomyelin (SM)	↑	A deregulation of sphingolipid metabolism is strongly linked to AD [89] and changes in SM level have been reported in AD brain tissue [82,85–87]. The vitamin B12-induced elevation of SM might be beneficial in respect to AD as SM has been shown to decrease Aβ production, possibly due to inhibition of γ-secretase activity [91]. Additionally, soluble Aβ peptides increase the activity of neutral and acid sphingomyelinases [90,91], downregulating SM level and increasing ceramide level, known to increase Aβ production [43–45].

The observed decrease in β- and γ-secretase activity in the cellular AD-model after vitamin B12 treatment further substantiates—beside our findings that PCaa as well as plasmalogen level are elevated in vitamin B12 treated APPswe expressing cells—that key findings of our study can be also found in the AD-model. The human neuroblastoma cell line SH-SY5Y is typically utilized to investigate biochemical pathways and mechanisms involved in AD [116–119]. However, as a potential caveat our study is based on cell culture experiments and further studies in e.g., 5xFAD mouse models as a typical transgenic AD mouse model [27,120] should be performed to address the impact of vitamin B12 on lipid changes related to AD in vivo.

Furthermore, we would like to emphasize that the observed changes ranged from 10–60%, which might be assumed to be mild to moderate changes. However, changes with the same effect strength were observed for altered lipids in human post mortem AD brains, e.g., plasmalogens were reported to be decreased by 5–25% [26].
